# A Protein Phosphorylation Threshold for Functional Stacking of Plant Photosynthetic Membranes

**DOI:** 10.1371/journal.pone.0010963

**Published:** 2010-06-04

**Authors:** Rikard Fristedt, Pontus Granath, Alexander V. Vener

**Affiliations:** Department of Clinical and Experimental Medicine, Linköping University, Linköping, Sweden; Purdue University, United States of America

## Abstract

Phosphorylation of photosystem II (PSII) proteins affects macroscopic structure of thylakoid photosynthetic membranes in chloroplasts of the model plant Arabidopsis. In this study, light-scattering spectroscopy revealed that stacking of thylakoids isolated from wild type Arabidopsis and the mutant lacking STN7 protein kinase was highly influenced by cation (Mg^++^) concentrations. The stacking of thylakoids from the *stn8* and *stn7stn8* mutants, deficient in STN8 kinase and consequently in light-dependent phosphorylation of PSII, was increased even in the absence of Mg^++^. Additional PSII protein phosphorylation in wild type plants exposed to high light enhanced Mg^++^-dependence of thylakoid stacking. Protein phosphorylation in the plant leaves was analyzed during day, night and prolonged darkness using three independent techniques: immunoblotting with anti-phosphothreonine antibodies; Diamond ProQ phosphoprotein staining; and quantitative mass spectrometry of peptides released from the thylakoid membranes by trypsin. All assays revealed dark/night-induced increase in phosphorylation of the 43 kDa chlorophyll-binding protein CP43, which compensated for decrease in phosphorylation of the other PSII proteins in wild type and *stn7*, but not in the *stn8* and *stn7stn8* mutants. Quantitative mass spectrometry determined that every PSII in wild type and *stn7* contained on average 2.5±0.1 or 1.4±0.1 phosphoryl groups during day or night, correspondingly, while less than every second PSII had a phosphoryl group in *stn8* and *stn7stn8*. It is postulated that functional cation-dependent stacking of plant thylakoid membranes requires at least one phosphoryl group per PSII, and increased phosphorylation of PSII in plants exposed to high light enhances stacking dynamics of the photosynthetic membranes.

## Introduction

Plant chloroplasts contain extremely large and well organized photosynthetic thylakoid membranes with highly stacked membrane layers [1], [2] enriched in photosystem II (PSII), which uses light energy to oxidize water and produce oxygen [3]. The flattened sacks of thylakoid membranes form multiple stacks, called grana, which allow fitting of the enormous membrane surface inside a chloroplast. The area-to-volume ratio of plant thylakoids is 70–500 times bigger than that for a sphere-like vesicle, resulting in the increased ability of the chloroplasts to capture light energy [4]. Thus, thylakoids of higher plants are organized in two distinct domains: 80% of the membrane comprises the grana stacks that are connected by non-appresed stroma lamellae membranes [5], [6]. The grana stacking of thylakoids is important for several regulatory processes of plant photosynthesis, like balancing of the excitation energy between the two photosystems and thermal dissipation of excess excitation energy via non-photochemical quenching [4], [6].

We recently demonstrated that phosphorylation of PSII proteins in the model plant *Arabidospis thaliana* affects macroscopic structure of thylakoids. The *Arabidopsis stn8* and *stn7stn8* mutants defective in phosphorylation of PSII have grana stacks that are markedly bigger than in thylakoids of wild type plants [7]. This enhanced grana size, visualized in the leaves of the mutant plants by electron microscopy, obstructs lateral migration of the PSII reaction centre protein D1 and of the processing protease FtsH between the stacked and unstacked membrane domains, and suppresses turnover of damaged D1 in the leaves exposed to high light [7]. Notably, the length of grana stacks in mature chloroplasts of all studied plant species is rather constant, about 400 nm [5], and larger grana diameter could be disadvantageous for lateral protein diffusion processes [8], like it has been demonstrated in *Arabidopsis* mutants deficient in light-induced phosphorylation of PSII [7]. Formation of thylakoid grana depends on the complex interplay of physicochemical forces of attraction and repulsion [4] The *in vitro* studies clearly demonstrated that electrostatic forces control the stacking and unstacking of isolated thylakoid membranes [9], [10], [11], [12]. Protein phosphorylation occurs at the outer surface of thylakoid membranes [13], [14] and contributes to the total negative charge of the membrane surface [4].

Thylakoid protein phosphorylation is mediated by a redox-sensitive regulatory system reflecting different light and other environmental conditions [14], [15], [16], [17]. The recent years revealed two major protein kinases involved in these phosphorylation events in *Arabidopsis*. The thylakoid associated Ser-Thr kinase STN7 is essential for phosphorylation of light harvesting complex polypeptides, LHCII, of the minor light harvesting protein CP29 and of TSP9, a soluble protein involved in regulation of light harvesting [17], [18], [19], [20], [21]. The phosphorylation of PSII core proteins is mediated through an ortholog of STN7 called STN8 kinase [18]. STN8 is involved in phosphorylation of the D1, D2, CP43 and PsbH proteins of PSII, and the calcium-sensing receptor (CaS) protein [22], [23]. Phosphorylation of LHCII and PSII core proteins serves important, while different adaptive functions. The first is crucial for the photosynthetic state transitions when reversible phosphorylation of mobile LHCII modulates its association with either PSII or photosystem I, allowing for optimal usage of excitation energy at low light intensities [17], [21]. Differential phosphorylation of LHCII-PSII linker protein CP29 [24], [25] and the TSP9 protein, localized at the LHCII-PSII interface, also regulate state transitions [20], [26], [27]. Phosphorylation of PSII core proteins is important for sustained photosynthesis in plants exposed to high light and for turnover of the light-damaged D1 core protein of PSII [7], [16], [28].

Phosphorylation of LHCII and PSII proteins is regulated according to dark and light conditions. However, LHCII is quickly phosphorylated under low light and dephosphorylated in darkness, while the PSII core has a more stable phosphorylation during the photoperiod [13]. Moreover, under high light conditions phosphorylation of PSII core proteins is increased while that of LHCII is drastically reduced [13], [15], [21], [29], [30]. The relationship between the dynamic light-dependent phosphorylation of thylakoid proteins on the one hand and stability of thylakoid grana stacks in plant chloroplasts [5], [8] on the other hand is not presently resolved. Particularly, there are no quantitative data on the PSII phosphorylation in plant leaves during day/night transitions in relation to thylakoid stacking. Moreover, the threshold of the PSII protein phosphorylation between the *stn8* and *stn7stn8* protein kinase mutants with the abnormal grana size of the tylakoids and the wild type and *stn7 Arabidopsis* plants with the normal membrane organization [7] remains to be determined.

In this study we analyze the phosphorylation of PSII proteins in leaves of *Arabidopsis thaliana* wild type and *stn7*, *stn8*, and *stn7stn8* mutants at different photoperiodic time points and during prolonged darkness. For comprehensive analysis of the *in vivo* protein phosphorylation under various light/dark conditions we use three different complementary techniques: western blotting with two different anti-phosphothreonine antibodies; Diamond ProQ staining of phosphorylated proteins; and quantitative mass spectrometry of non-phosphorylated and phosphorylated peptides released from the thylakoid membranes. Furthermore, we also use light-scattering spectroscopy to monitor changes in thylakoid membrane structure depending on the protein phosphorylation state and varying concentrations of MgCl_2_, a salt important in maintaining grana structures in isolated thylakoid membranes [9], [10]. Our data reveal the state of PSII protein phosphorylation during the photoperiod and demonstrate that phosphorylation of at least one protein per every PSII is required for the natural Mg^++^-dependent stacking of *Arabidopsis* thylakoid membranes.

## Results and Discussion

### Effects of MgCl_2_ on stacking of isolated thylakoid membranes

**Figure 1:**
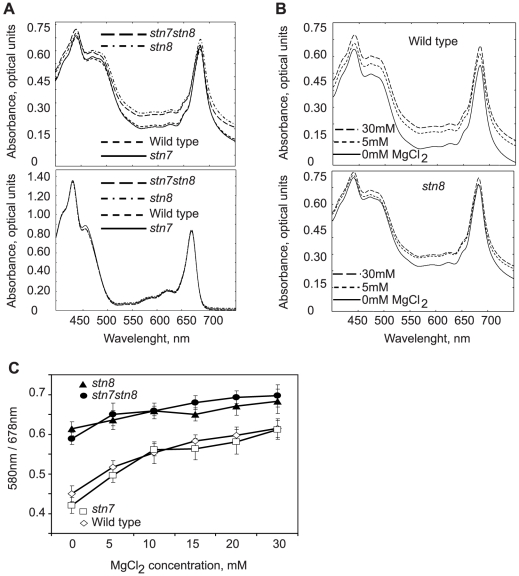
Absorbance spectra of thylakoid membranes and effects of MgCl_2_ titration on light scattering. A, the spectra of native thylakoid suspensions from wild type, *stn7*, *stn8* and *stn7stn8*, as indicated, in the presence of 5 mM MgCl_2_. Representative spectra are superimposed in the upper panel. Lower panel demonstrates spectra of the same thylakoid membranes after extraction with 80% acetone. B, light-scattering changes as a function of MgCl_2_ titration: the representative spectra at 0, 5 and 30 mM MgCl_2_, as indicated, are shown for wild type (upper panel) and *stn8* (lower panel) thylakoid membranes. C, titration of the light-scattering changes in the thylakoid membranes from wild type, *stn7*, *stn8* and *stn7stn8*, as indicated, using 0, 5, 10, 15, 20 and 30 mM MgCl_2_ and normalization of absorbance at 580 nm to the 678 nm signal. Error bars represent S.D. of at least three independent experiments.

Thylakoid membranes carry net negative charges on their surfaces and electrostatic interactions control the stacking and unstacking of these membranes [4], [12]. The extent of the stacking of isolated thylakoids could be directly monitored via light scattering of their suspensions by recording the optical density at 580 nm [9], [10]. Furthermore, it had been shown by electron microscopy that chloroplasts isolated in media containing 5 mM MgCl_2_ retained stacked thylakoids, while chloroplasts isolated in low salt media lost their grana structure and had a reduced absorbance at 580 nm [9], [10]. The *stn7stn8* and *stn8* mutants of *Arabidopsis* are deficient in light-induced phosphorylation of PSII and have increased size of stacked thylakoid membranes compared to wild-type and *stn7* plants, as has been revealed by transmission electron microscopy [7]. Here, we analyzed the changes in the light-scattering properties of isolated thylakoid membranes by recording the absorbance of their suspensions in the region 400–750 nm. Figure 1A, upper panel, shows the characteristic spectra from wild type, *stn7*, *stn8* and *stn7stn8* membranes resuspended in a buffer containing 5mM MgCl_2_, to retain the native grana and stroma lamellae structures. It is clearly seen that the scattering properties of the thylakoids are different between wild type and *stn7* on one hand and *stn8* and *stn7stn8* on the other hand. The difference is clear due to the higher absorbency of the *stn8* and *stn7stn8* membranes in the yellow/orange range of 570–620 nm. Notably, Figure 1A, lower panel, shows the almost identical absorbance spectra of chlorophylls and other pigments after their extraction by acetone from the wild type and all the mutant membranes. This figure also displays the increase in the absorbance of the major chlorophyll peaks after their extraction from the native membranes (compare with Figure 1A, upper panel).

In order to confirm that increase in absorbance at 570–620 nm for the *stn8* and *stn7stn8* mutants (Figure 1A) was a consequence of enhanced thylakoid membrane stacking [7], [9], [10] we performed titration of the membrane suspensions with 0, 5, 10, 20 and 30 mM MgCl_2_. Figure 1B shows the influence of MgCl_2_ on the spectral properties of thylakoids from wild type and *stn8*. The wild type and *stn7* membranes followed the same pattern with high increase in absorbance in the yellow/orange spectral range upon increasing concentrations of MgCl_2_ (Figure 1C). The higher absorbance at increasing concentration of MgCl_2_ was a consequence of salt-mediated stacking of thylakoid membranes [9]. However, *stn8* and *stn7stn8* showed a much higher absorbance of isolated thylakoids already at 0 mM MgCl_2_ and that absorbance was less affected by MgCl_2_ (Figure 1C). In order to quantify the increase in absorbance at 580 nm for *stn8* and *stn7stn8* versus wild type and *stn7* we normalized the signals at 580 nm to those at 678 nm and generated the graph in Figure 1C. This graph shows that wild type and *stn7* have an overall lower absorbance value at 580 nm and are more influenced by MgCl_2_ titration than the *stn8* and *stn7stn8* membranes.

**Figure 2:**
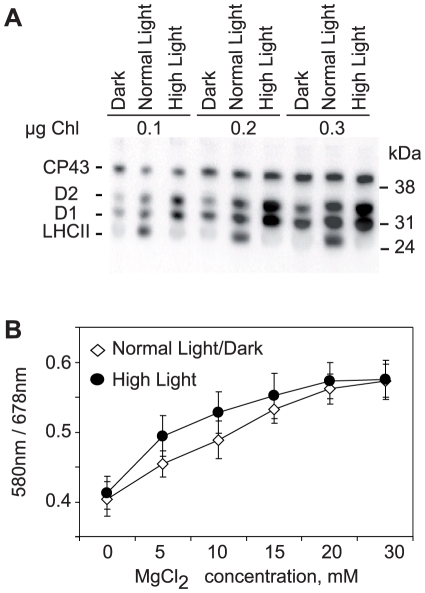
High light induced increase in PSII phosphorylation and its effect on MgCl_2_–dependence of thylakoid membrane light scattering. A, thylakoid membrane proteins from wild type plants adapted to dark, or exposed to normal or high light for 3 hours were separated on SDS-PAGE and immunoblotted with anti-phosphothreonine antibody from Zymed laboratories. The samples were loaded at three different chlorophyll concentrations, as indicated. Positions of the phosphorylated thylakoid proteins and of the molecular mass markers are indicated. B, titration of the light-scattering changes in thylakoid membranes from the leaves exposed to normal or high light, as indicated, using 0, 5, 10, 15, 20 and 30 mM MgCl_2_ and normalization of absorbance at 580 nm to the 678 nm signals. Error bars represent S.D. of at least three independent experiments.

As a next step we did analysis of Mg^++^-dependent folding of thylakoid membranes isolated from wild type plants exposed to three hours of high light treatment. Immunoblotting of the thylakoid proteins from the leaves exposed to darkness, normal light and high light demonstrated the high-light-induced rise in phosphorylation of D1 and D2 proteins of PSII (Figure 2A). The spectroscopic analysis of the isolated thylakoids showed that absorbance of the membranes from dark, normal and high light exposed plants was similar, when the membranes were resuspended in the absence of MgCl_2_. However, the higher phosphorylation of PSII core proteins from high light exposed plants resulted in the steeper absorbance rise when the membranes were titrated with MgCl_2_ (Figure 2B). At the higher levels of MgCl_2_ the dark, normal and high light acclimated membranes had similar absorbance at 580 nm, as a consequence of saturated Mg^++^-induced stacking. Thus, if the reduced phosphorylation of PSII in the *stn8* and *stn7stn8* mutants diminishes Mg^++^-dependence of thylakoid folding (Figure 1), increased phosphorylation of PSII in the high-light-treated leaves of wild type makes folding of thylakoids more receptive to lower concentrations of Mg^++^ (Figure 2).

Phosphorylated PSII complexes are facing each other in the compressed thylakoid grana membranes [8], [31], so the negatively charged phosphoryl groups repel each other. When Mg^++^ ions are present in the medium the phosphoryl groups exposed at the membrane surface are neutralized by these cations, which can also bridge the adjacent membrane layers and thus stabilize the grana structures. In the *stn8* and *stn7stn8* mutants defective in phosphorylation of PSII the adjacent membranes have a lower negative charge and are more stacked [7], thus the Mg^++^ ions induce only minor additional structural changes in the thylakoids. These data demonstrate that a certain stable level of PSII phosphorylation is important for maintenance of optimal cation-dependent stacking of plant thylakoid membranes.

### Photoperiodic changes in thylakoid protein phosphorylation

**Figure 3:**
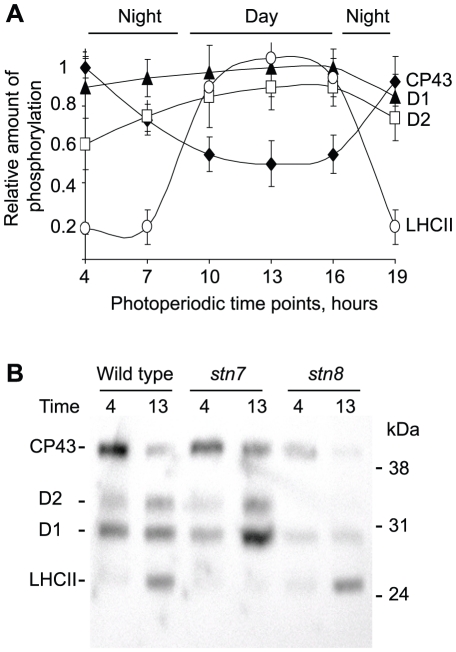
Thylakoid protein phosphorylation during the photoperiod. A, relative changes in the PSII and LHCII protein phosphorylation determined by immunoblotting analyses of SDS-PAGE separated thylakoid proteins with anti-phosphothreonine antibody. Thylakoids were prepared from leaves of the wild type plants harvested at the indicated time points during night or day (4, 7, 19 are the hours of the dark phase and 10, 13, 16 are the hours of the light phase of the photoperiod). Error bars represent S.D. of at least three independent experiments. B, immunoblot of thylakoid proteins from the *stn* mutants and wild type, as indicated, with anti-phosphothreonine antibody from Zymed Laboratories. Positions of the phosphorylated thylakoid proteins and of the molecular mass markers are indicated. Time points correspond to the highest and lowest phosphorylation of CP43 in the wild type plant leaves: after 12 hours of darkness (time point 4) and after 5 hours of light (time point 13).

To address the question of protein phosphorylation dynamics in thylakoid membranes of *Arabidopsis thaliana* we harvested leaves at different time points of the plant growth photoperiod (16 hours dark and 8 hours light). To preserve the *in vivo* protein phosphorylation we isolated thylakoid membranes in the presence of NaF, an inhibitor of thylakoid protein phosphatases [32]. The phosphorylation state of major thylakoid phosphoproteins was analyzed by immunoblotting with anti-phosphothreonine antibody (Figure S1). In agreement with the earlier studies [13] we observed significant increase in LHCII phosphorylation during the light period and almost complete dephosphorylation during night (Figure 3A). To the contrary, the phosphorylation state of the D1 and D2 proteins of PSII was rather stable during the whole photoperiod, with some increase in phosphorylation during the hours of light exposure (Figure 3A). Strikingly, CP43 phosphorylation was regulated in the opposite way: it was higher during the night and approached its lowest levels during the middle of the light period (Figure 3A). Quantification of phosphorylation levels for CP43, D1, D2 and LHCII proteins (Figure 3A) revealed the most representative time points for night and day at 4 am and 1 pm (time point 13 in the Figure 3A), correspondingly. We also assayed phosphorylation states of the thylakoid proteins in the *stn7* and *stn8* mutants at these time points representative for night and day (Figure 3B). The light-dependent phosphorylation of LHCII was absent in *stn7*, while phosphorylation of the D1 and D2 proteins was significantly reduced in *stn8*, in agreement with the previous studies [18], [22]. In respect to the night-dependent rise in phosphorylation of CP43, the *stn7* mutant followed the same pattern as in the wild type, while *stn8* showed a very low dark-induced CP43 phosphorylation as compared to the wild type and *stn7* plants (Figure 3B). A distinct increase in CP43 phosphorylation observed after exposure of the wild type plants to darkness occurred in parallel to the dramatic decrease in phosphorylation of LHCII and some moderate reduction in phosphorylation of the D1 and D2 proteins. Thus, phosphorylation of CP43 during the light to dark transition in *Arabidopsis* is regulated opposite to that of the other PSII and LHCII proteins.

### Phosphorylation dynamics of PSII proteins in darkness

**Figure 4:**
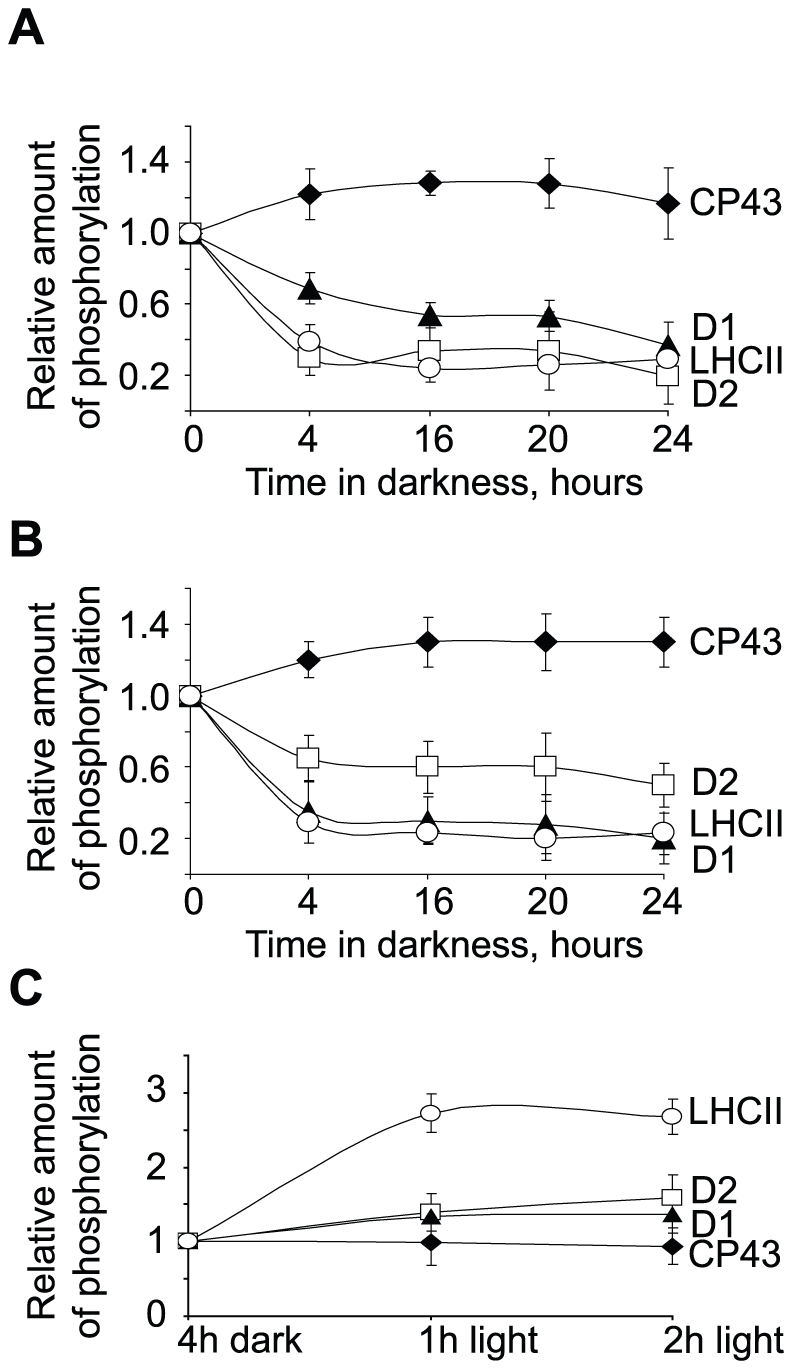
Phosphorylation of thylakoid proteins during prolonged darkness or during dark/light transitions not related to photoperiod. Relative quantification of the PSII and LHCII protein phosphorylation in thylakoid membranes separated by SDS-PAGE and immunoblotted with anti-phosphothreonine antibodies from either Zymed Laboratories (A and C) or Cell Signaling (B). A and B, thylakoids were isolated from the wild type plants exposed for 4 hours to normal light (the time point 0) and then transferred to darkness for 4, 16, 20 and 24 hours, as indicated. C, wild type plants were exposed to normal light for 3 hours, then incubated for 4 hours in darkness (the time point 4h dark) and re-exposed to normal light for 1 or 2 hours, as indicated. Error bars represent S.D. of at least three independent experiments.

To make a detailed examination of PSII phosphorylation state in darkness, with particular attention to the unexpected increase in CP43 phosphorylation level, we studied protein phosphorylation during prolonged dark incubation of plants using several complementary assays. Thylakoid membranes were isolated from leaves of Arabidopsis plants exposed to 4 hours of normal growth light, the time point that we used as a control, or from the plants which were kept in darkness for 4, 16, 20 and 24 hours. We analyzed phosphorylation levels of thylakoid proteins using two complementary anti-phosphothreonine antibodies from Zymed Laboratories and from New England Biolabs (Cell Signaling), which differ from each other in sensitivity against major thylakoid phosphoproteins [20], [29], [33]. Phosphorylation of LHCII and of two PSII core proteins, D1 and D2, decreased and stayed at different steady state levels during prolonged incubation in darkness (Figure S2 and Figure 4A,B). These changes in phosphorylation patterns were detected with both Zymed (Figure S2A and Figure 4A) and Cell Signaling (Figure S2B and Figure 4B) antibodies. However, phosphorylation of the CP43 protein was increased in the plants kept in darkness (Figure S2A,B and Figure 4A,B). These data were statistically significant according to calculations of the phosphoryaltion levels from four different experiments for each anti-phosphothreonine antibody. We also used protein specific antibodies against CP43 (Figure S2A, lower panel) and D1 (Figure S2B, lower panel) to verify even sample loading, as well as stable protein expression during prolonged incubation of plants in darkness. No significant change in expression of CP43 during 20 hours of dark incubation was observed (Figure S2A, lower panel), however, the increase in CP43 phosphorylation was already seen after 4 hours of darkness and it remained stable for up to 20 hours of dark incubation. Taken together, these results revealed a substantial up-regulation of CP43 phosphorylation in plant leaves subjected to long incubation in darkness, and were consistent with the increase of CP43 phosphorylation during night phase of usual photoperiod (Figure 3).

To investigate if the observed changes in protein phosphorylation patterns depend on photoperiod or only the light/dark transitions we exposed *Arabidopsis* plants to 3 hours of light (normal photoperiod), transferred plants to darkness for 4 hours, and then exposed them to light for 1 or 2 hours. The thylakoid membranes were isolated from the leaves harvested at these time points and analyzed by western blotting using anti-phosphothreonine antibody (Figure S2C). The quantitative analysis of phosphorylation changes for CP43, D1, D2 and LHCII proteins under these conditions is shown in Figure 4C. LHCII from the light-exposed plants had a high phosphorylation state, but was rapidly dephosphorylated upon the transition to darkness and phosphorylated again when the plants were re-exposed to light (Figure 4C). The changes in phosphorylation of the PSII core proteins were more complex. Phosphorylation of D1 and D2 decreased, while phosphorylation of CP43 increased after the transition to darkness for 4 hours. However, none of these three PSII proteins changed its phosphorylation state significantly during the following exposure of the plants to light for 2 hours (Figure 4C).

**Figure 5:**
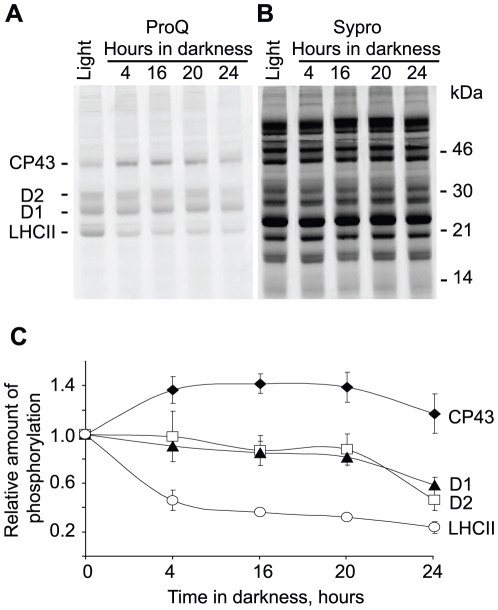
Phosphorylation of thylakoid proteins during prolonged darkness assayed with ProQ phosphoprotein stain. A, ProQ phospho-stain analysis of SDS-PAGE separated thylakoid proteins from wild type plants exposed to normal light for 4 hours (Light) and then transferred to darkness for 4, 16, 20 and 24 hours, as indicated. B, Sypro total protein stain on the same gel as in A. Positions of the phosphorylated thylakoid proteins and of the molecular mass markers are indicated. C, relative quantification of the protein phosphorylation from the gels stained like in A and B, at the corresponding time points of plant incubation in darkness. Error bars represent S.D. of at least three independent experiments.

To characterize the changes in endogenous phosphorylation of the PSII proteins by an independent analytical technique we also used Pro-Q Diamond phosphoprotein gel stain in combination with Sypro Ruby total protein gel staining. The combination of these two staining techniques provides a measure of the relative phosphorylation level of the protein in each band on the gel. The Pro-Q staining showed a clear difference in the directions of phosphorylation change for CP43 compared to D1, D2 and LHCII in the plants transferred to darkness (Figure 5A). The Sypro Ruby total protein stain demonstrated that the amount and expression of all the analyzed proteins was stable during the dark incubation (Figure 5B). The phospho-stain data and the total protein-staining allowed calculation of a relative phosphorylation ratio for the CP43, D1, D2 and LHCII proteins (Figure 5C). CP43 phosphorylation was up-regulated during the dark-transition, while phosphorylation of D1, D2 and, most obviously, LHCII was down regulated in the dark. These data confirmed the results obtained with anti-phosphothreonine antibodies and reinforced an important finding, namely that CP43 phosphorylation in darkness is regulated differently from that of the D1 and D2 core proteins of PSII or LHCII polypeptides.

### Protein phosphorylation in the kinase mutants during prolonged darkness

**Figure 6:**
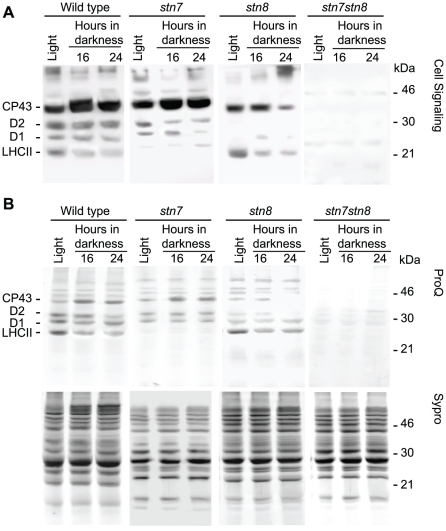
Protein phosphorylation patterns in the *stn* mutants. A, immunoblotting analysis of SDS-PAGE separated thylakoid proteins from wild type, *stn7*, *stn8* and *stn7stn8* mutant plants, as indicated, with anti-phosphothreonine antibody from Cell Signaling. B, ProQ phospho-stains and Sypro total protein stains, as indicated, of SDS-PAGE separated thylakoid proteins from the same samples as in A. The numbers 16 and 24 correspond to the time of dark incubation in hours.

In order to investigate how the PSII phosphorylation dynamics is affected during prolonged incubation in darkness in the absence of either STN7 or STN8 kinase, or both of them, we used the *stn7*, *stn8* and *stn7stn8* mutants. To this end we did analyses using both western blotting and ProQ phospho-stain. First, the thylakoid samples isolated from mutants and wild type plants exposed to growth light for 4 hours or darkness for 16 and 24 hours were immunoblotted using anti-phosphothreonine antibody. Wild type, as well as *stn7* plants clearly showed increase in CP43 phosphorylation under dark conditions (Figure 6A). No significant increase in CP43 phosphorylation in the dark-exposed *stn8* was observed, and phosphorylation of this protein in *stn7stn8* was not detected (Figure 6A). The similar results were obtained when the phosphorylation states of thylakoid proteins from the mutant plants were assayed using ProQ phospho-stain (Figure 6B). These results demonstrated that STN7 is not directly involved in the dark-induced phosphorylation of CP43. In the *stn8* mutant phosphorylation of CP43 at all time points was lower than in the wild type or *stn7* plants (Figure 6A,B), while no any significant phosphorylation was detected in *stn7stn8*, which demonstrated requirement of both STN7 and STN8 for quantitative phosphorylation of the thylakoid proteins [7], [17].

Our results reveal a complex regulatory process for CP43 phosphorylation, which includes both dark- and photoperiod-dependence. We determine that STN8 is at least partially responsible for the dark-induced phosphorylation of CP43, since its remaining phosphorylation in *stn8* membranes is low during either light to dark transitions or photoperiod. Besides the redox regulation [29], [34], [35], thylakoid protein phosphorylation is also controlled at the substrate level by conformational changes of membrane proteins in response to light [36]. Furthermore, it has been shown that CP43 phosphorylation depends on the light-induced structural changes in this protein [37]. This work demonstrated that illumination of PSII cores affected the conformation of CP43 and expose of its N-terminus, containing the phosphorylation site, to the enzymes such as kinases and proteases [37]. We present evidence that phosphorylation of CP43 is decreased during the day. Accordingly, the light-induced conformational change in CP43 may be beneficial to the phosphatase accountable for its dephosphorylation. The enzymatic process responsible for dephosphorylation of CP43 has been characterized [32], while the corresponding protein phosphatase is still unknown and it is clearly distinct from the PPH1 phosphatase, which does not dephosphorylate the PSII core proteins [38].

### Quantitative analysis of PSII protein phosphorylation

**Figure 7:**
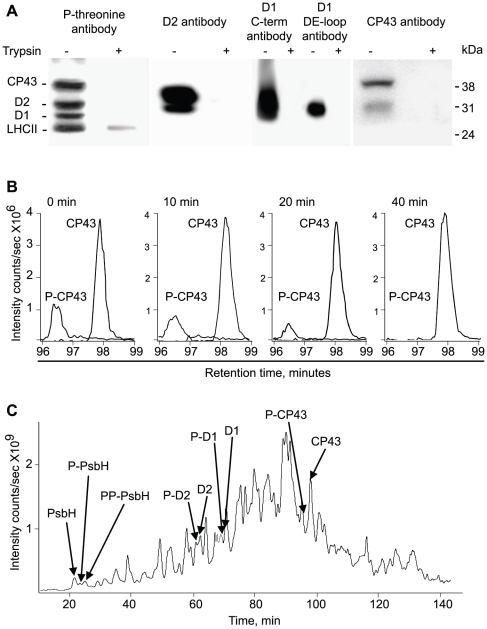
Quantification of PSII core protein phosphorylation using LC-MS. A, immunoblotting analyses of thylakoid membrane proteins from wild type plants with antibodies against CP43, D2, D1 and phosphothreonine before (−) and after (+) trypsin treatment, as indicated. B, LC-MS extracted ion chromatograms of the phosphorylated and non-phosphorylated N-terminal peptide from the CP43 protein after incubation of the peptide mixture with alkaline phosphatase for different time, as indicated. The ratios of phosphorylated to non-phosphorylated peptide intensities at each time point were used for calculations of CP43 phosphorylation level. C, LC-MS profile of total ion chromatogram with indication of the elution times for peptide/phosphopeptide pairs from PSII core proteins. Experimental procedures as in A, B and C were done for wild type and *stn7*, *stn8*, *stn7stn8* plants from normal light (4 hours of light) and dark (12 hours of darkness).

To quantify phosphorylation changes during day and night in the wild type, *stn7*, *stn8* and *stn7stn8* plants we used high performance liquid chromatography coupled with mass spectrometry. Thylakoid membranes isolated from wild type and mutant plants were subjected to proteolytic shaving by trypsin to remove the surface exposed phosphorylated peptides for vectorial proteomics [13], [14], [39]. This approach depends on complete proteolytic cleavage of the peptides from the membrane proteins, particularly when used quantitatively. We assayed proteolytic cleavage of the thylakoid proteins using western blotting. Figure 7A shows the results of analyses of thylakoid proteins with antibodies against CP43, D2, D1 C-terminus, D1 DE-loop and anti-phosphothreonine antibodies before and after trypsin treatment. These data demonstrate that the proteolytic cleavage of the membrane proteins was very efficient and no intact protein signal was detected after 3 hours of trypsin digestion.

HPLC in conjugation with Electro Spray Ionization Mass Spectrometry (ESI-MS) allows resolution and detection of the major phosphorylated peptides and their non-phosphorylated counterparts for determination of the phosphorylation stoichiometry for PSII proteins [13]. In the present work we improved this earlier described method. To obtain accurate data on quantitative phosphorylation of PSII proteins we introduced a normalization procedure that takes into account the differences in ionization and in signal intensities for phosphorylated and corresponding non-phosphorylated peptides [40]. We determined the flyability ratio [40] for each particular peptide/phosphopeptide pair and used these ratios to correct the signal intensities of the phosphorylated peptides for absolute phosphorylation stoichiometry measurements. This approach is built on the fact that the decrease in the amount of phosphorylated peptide during enzymatic dephosphorylation is equal to increase in the amount of its non-phosphorylated cognate peptide. To generate samples with varying phosphorylation stoichiometry the thylakoid peptide mixtures were treated with alkaline phosphatase under controlled conditions: the aliquots were taken at 0, 10, 20 and 40 min of the phosphatase treatment. Figure 7B shows the ion intensity signals of phospho (678.4^2+^) and de-phospho (638.4^2+^) forms of the CP43 peptide (Ac-TLFNGTLALAGR) at different time points and decrease in amount of phosphorylation upon the phosphatase treatment. The similar measurements were done for peptide/phosphopeptide pairs from the D1, D2 and PbsH proteins and their flyability ratios were calculated using 0, 10, 20, 40 min time points (Table 1). These ratios were used to correct the signal intensities of the phosphorylated peptides in measurements of the protein phosphorylation states by calculation of peptide/phosphopeptide ratios for each particular PSII protein (see below and Table 2).

**Table 1:** Flyability ratios for PSII peptide ions and their phosphorylated cognates.

Protein	Peptide/phosphopeptide sequence	z	Flyability ratio
CP43	*Ac*-TLFNGTLALAGR/*Ac*-tLFNGTLALAGR	2+	1.23±0.14
PsbH	ATQTVEDSSR/AtQTVEDSSR	2+	0.94±0.17
PsbH	ATQTVEDSSR/AtQtVEDSSR	2+	0.89±0.13
PsbH	AtQTVEDSSR/AtQtVEDSSR	2+	1.14±0.21
D1	*Ac*-TAILER/*Ac*-tAILER	1+	1.17±0.11
D2	*Ac*-TIALGK/*Ac*-tTIALGK	1+	1.29±0.10

The thylakoid peptide mixtures were treated with alkaline phosphatase for 0, 10, 20 and 40 min to obtain samples with varying degrees of phosphorylation. The signal intensity of each peptide ion was obtained using LC-MS and flyability ratio for each peptide/phosphopeptide pair was determined as (IB−IA)/(IpA−IpB), were IpA and IA are the ion intensities of the phosphorylated and non-phosphorylated peptides at 0 min time point, and IpB and IB are the corresponding ion intensities at the 10, 20 or 40 min time points. An average flyability ratio for each peptide/phosphopeptide pair was calculated from the data corresponding to all time points of alkaline phosphatase treatment. *Ac*-, N-terminal acetylation; lower case t, phosphorylated threonine residue in peptide sequence; z, the charge state of the peptide ion.

**Table 2:** In vivo phosphorylation stoichiometry (% of phosphorylation) for the PSII core proteins from the leaves of wild type, *stn7*, *stn8* and *stn7stn8* mutant plants harvested during day or night.

	Wild type		*stn8*	
	12h Dark	4h Light	12h Dark	4h Light
Protein	% of phosphorylation		% of phosphorylation	
P-CP43	49±12	34±13	16±8	13±7
P-D1	27±12	36±15	12±9	14±8
P-D2	33±11	42±12	10±6	10±4
P-PsbH	29±9	24±12	11±8	9±8
PP-PsbH	N.D.	54±14	N.D.	N.D.

	*stn7*		*stn7stn8*	
	12h Dark	4h Light	12h Dark	4h Light
Protein	% of phosphorylation		% of phosphorylation	
P-CP43	44±13	36±9	N.D.	N.D.
P-D1	29±12	35±8	N.D.	N.D.
P-D2	33±13	44±7	<3*	<4*
P-PsbH	29±15	22±8	<4*	<4*
PP-PsbH	N.D.	57±9	N.D.	N.D.

N.D. - not determined.

*The PsbH (AtQTVEDSSR) and D2 (Ac-tIALGK) phosphopeptide signals were detected in the samples from *stn7stn8* only in one or two out of three different experiments.

The phosphorylation state of each protein was determined from signal intensities of peptide and phosphopeptide ion pairs corrected using corresponding flyability ratios from the Table 1. Data are the average from three experiments in each condition and three LC-MS runs for every experiment.

We performed LC-MS analyses of the peptide mixtures from thylakoids isolated from leaves harvested during the day or night. The typical chromatogram is shown in Figure 7C with indication of elution times for the phosphorylated and non-phosphorylated peptides from the PSII core proteins. Phosphorylation stoichiometry of these proteins under day or night conditions was determined in the wild type, *stn7*, *stn8* and *stn7stn8* plants. The results are presented in Table 2. Phosphorylation state of the PSII core proteins in leaves of the wild type plants harvested during day or night was rather stable. In agreement with the earlier study [13] at least one third of CP43, D1, D2 or PsbH was phosphorylated during the dark or light phases of the photoperiod. Only appearance of the doubly phosphorylated form of PsbH [13], [22] was strictly light-dependent (Table 2). Account of the differences in ionization and in signal intensities for phosphorylated and corresponding non-phosphorylated peptides and use of the flyability constants (Table 1) allowed for detection of increase in phosphorylation of the CP43 protein during night (Table 2). This quantitative MS analysis supported our immunoblotting and staining results: the extent of CP43 phosphorylation in the wild type increased from 34% during the day to 49% during the night, which corresponded to a relative 44% increase in the phosphorylation upon day to night transition.

Phosphorylation of the PSII core proteins during day or night in leaves of *stn7* was very similar to that in wild type (Table 2), showing that the light-regulated kinase STN7 [17], [18], [35] is not directly involved in control of PSII phosphorylation during dark or light phases of the photoperiod. On the contrary, phosphorylation of the CP43, D1 and D2 core proteins in *stn8* was 3 to 4 times lower than in wild type or *stn7* plants and no increase in phosphorylation of the CP43 protein during night was found (Table 2). No significant phosphorylation of the PSII core proteins was detected in leaves of the *stn7stn8* double mutant plants. Using the MS approach we found doubly phosphorylated PsbH corresponding to about 50% of total PsbH in light-exposed leaves of wild type and *stn7*, but it was totally absent in *stn8* plants. On average at least one third of each of the PSII core protein was always phosphorylated during photoperiod in wild type and *stn7*, while in *stn8* plants this phosphorylation was three times lower (Table 2).

Quantitative data from Table 2 and account of the equivalent molar amounts of the CP43, D1, D2 and PsbH proteins in the PSII core allowed calculations of average number of phosphoryl groups per PSII in leaves of the wild type, *stn7* and *stn8* plants during day or night. In the wild type *Arabidopsis* every PSII contained 1.38±0.11 or 2.44±0.13 phosphoryl groups during night or day, respectively. In the *stn7* mutant these numbers were very similar: 1.35±0.13 or 2.51±0.08, correspondingly. However, in the *stn8* mutant only 0.49±0.06 phosphoryl groups per PSII were present during night and 0.46±0.07 groups during the day. Phosphorylation of PSII during either day or night in the *stn7stn8* plants was much lower (Table 2).

### Phosphorylation of PSII and thylakoid stacking

**Figure 8:**
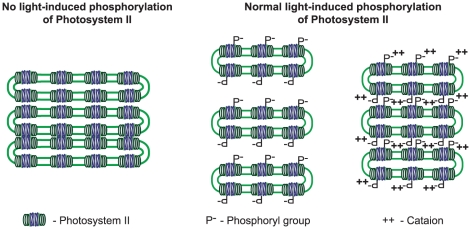
A model for phosphorylation- and cation-dependent stacking of plant photosynthetic membranes. Deficiency in light-induced phosphorylation of PSII in the *stn8* and *stn7stn8* mutant plants cause enlargement of grana and loss of the cation-dependent stacking of thylakoids (left). Presence of at least one phosphoryl group per PSII in the wild type thylakoids causes electrostatic repulsion of the adjacent grana membranes (center) and makes their stacking highly cation-dependent (right).

It had been suggested that the physiological significance of PSII core protein phosphorylation is in regulation of the repair cycle of photodamaged PSII: phosphorylation facilitates migration of damaged PSII centers from grana to stroma thylakoid regions, were the damaged D1 protein is dephosphorylated, degraded and exchanged to a newly synthesized copy [28], [41]. The thylakoid membranes contain big protein complexes and the high molecular ratio of proteins to lipids makes the thylakoids crowded. In the grana region, where PSII complexes are densely packed, movement of protein complexes may be considerably restricted [8]. This implies that the thylakoid membranes, especially in the grana stacks should be highly dynamic to allow for PSII migration [31]. Importantly, PSII-LHCII supercomplexes occupy about 50% of the grana space [31], so phosphorylation of PSII infers a massive negative change of the membrane surface and, consequently, repulsion of the adjacent grana stacks, as is schematically outlined in Figure 8. Quantitative mass spectrometry determined that every PSII in the wild type and *stn7* contained at least one phosphoryl group during either day or nigh, while less than every second PSII had a phosphoryl group in *stn8* and *stn7stn8*. This finding suggests that full-scale cation-dependent stacking of *Arabidopsis* thylakoid membranes (Figure 1) requires at least one phosphoryl group per PSII. The model in Figure 8 provides a simple explanation of all experimental data. In the mutant plants deficient in light-induced phosphorylation of PSII less than every second PSII has a phosphoryl group and the adjacent grana membranes are pressed to each other and enlarged [7] in the absence of the electrostatic repulsion. These thylakoids lost the cation-dependent stacking (Figure 1) and the lateral migration of membrane proteins, as well as the repair cycle of photodamaged PSII are retarded in *stn8* and *stn7stn8* mutant plants [7], [28]. The presence of more than one phosphoryl group per PSII in the wild type thylakoids causes repulsion of the adjacent grana membranes and their stacking is highly cation-dependent (Figure 8).

Increase in the PSII core protein phosphorylation in the wild type *Arabidopsis* under high light does not significantly affect the thylakoid membrane structure in the leaves [7], but makes the cation-dependent folding of isolated thylakoids more dynamic at physiological concentrations of Mg^++^ ions (Figure 2). This finding provides a possible link between the high-light-induced increase in PSII phosphorylation and facilitation in migration of damaged PSII centers from grana to stroma thylakoid regions during the PSII repair cycle [28], [41]. Notably, the recent study demonstrated more prominent photoinhibition of PSII in stacked than in unstacked thylakoids isolated from spinach, suggesting that unstacking of thylakoids has a crucial role in facilitating degradation of the photodamaged D1 under light stress [42].

### Conclusion

Using quantitative measurements of PSII core protein phosphorylation we determined the threshold of the phosphorylation required for the naturally selected optimal stacking of thylakoid membranes in *Arabidopsis*. The lowest phosphorylation state of the PSII core proteins D1, D2, CP43 and PsbH on average does not decrease below 30% in the wild type plants. Phosphorylation of these proteins in *stn7* is similar, while the absence of the LHCII phosphorylation in this mutant does not affect the thylakoid membrane structure in the leaves [7] or cation-dependent stacking of isolated thylakoids. Thus, LHCII phosphorylation does not influence macroscopic structure of thylakoids in *Arabidopsis*. Decrease in phosphorylation state of the PSII proteins to an average 12% in *stn8* mutant plants increases size of thylakoid grana in the leaves [7] and diminishes cation-dependent folding of isolated thylakoids. The similar effects are caused by almost complete absence of PSII core protein phosphorylation in the *stn7stn8* mutant of *Arabidopsis*.

The data indicate that the PSII core protein phosphorylation in plant thylakoids affects both macroscopic structure and dynamic properties of the photosynthetic membranes. We present evidence that CP43 phosphorylation is up-regulated during night, which partially compensates for the decrease in phosphorylation of other PSII core proteins during the dark phase of the photoperiod and could be important for the stable structure of plant thylakoids. The phosphorylation of CP43 in darkness is regulated differently from that of the D1 and D2 core proteins of PSII or LHCII polypeptides. PSII phosphorylation controls membrane stacking and lateral movements of the membrane components, which consequently may regulate processes such as PSII repair, plastoquinone diffusion and membrane biogenesis. These dynamic properties of thylakoid membranes allow plants to fine-tune photosynthesis, regulate the mechanisms of photoprotection and in general the adaptation to different environments. Elucidating the mechanisms involved in regulation of thylakoid structural flexibility we came closer to understanding of the functional properties of extremely large and organizationally complex photosynthetic membranes in the chloroplasts of higher plants.

## Materials and Methods

### Plant Material

*Arabidopsis thaliana* wild type (ecotype Columbia) plants, *stn7* (SALK 073254) [18], *stn8* (SALK 060869) [22], and double mutant *stn7stn8* [7], [18] in Columbia background used in this study were grown hydroponically [43] at 23°C, 65–70% relative humidity, photosynthetic flux of 120 µmol photons m^−2^ s^−1^ and photoperiod of 8 h light and 16 h dark. In the case of high light experiments photosynthetic flux of 900 µmol photons m^−2^ s^−1^ has been used.

### Isolation and characterization of thylakoids

Four-week-old plants were used for the preparation of chloroplasts and thylakoids. The thylakoid membranes were isolated from 4g of *Arabidopsis* leaves harvested at time points indicated. The leaves were homogenized in 20 ml of ice-cold 25 mM Tricine (pH 7.8), 330 mM sorbitol, 1mM EDTA, 10mM KCl, 0.15% bovine serum albumin, 4 mM sodium ascorbate, 7mM L-cysteine in a metal blender for four periods of 1 sec at high speed. The homogenate was immediately filtered through four layers of nylon mesh (20 µm pore size), and the filtrate was centrifuged for 3 min at 1000 g. The pellet was resuspended in the same buffer to wash the chloroplasts and centrifuged for 5 min at 1000 g. The chloroplast pellet was resuspended in 10 mM Tricine, 5 mM MgCl_2_, and 10 mM NaF and allowed to stand for 5 min in the dark on ice in order to lyse the chloroplasts. Following lysis, the thylakoids were pelleted by centrifugation for 5 min at 6000 g. To wash the thylakoids the pellet was resuspended in 100 mM sorbitol, 25 mM Tricine (pH 7.8), 5 mM MgCl_2_, 10 mM KCl, and 10 mM NaF and centrifuged for 5 min at 6000 g. The pellet was resuspended in a small volume of the same buffer. NaF was used as a phosphatase inhibitor in the buffers when phosphorylation was quantified. All procedures were made under weak green light at 4°C, and the sample was kept on ice throughout the whole process.

### Immunoblotting

Thylakoid membrane proteins were separated by SDS-PAGE (6% acrylamid stacking gel+14% separation gel+6M urea) and the proteins were transferred to a PVDF membrane (Immobilone, Millipore). For the anti-phosphothreonine antibodies, purchased from Zymed Laboratories Inc. or New England Biolabs (Cell Signalling), the membranes were blocked with 5% bovine serum albumin. For specific antibodies against the DE-loop in D1 protein [44], D1 C-terminus, residues 230–245 in the D2 protein and against CP43 protein [45], the blocking was done with 10% skimmed milk. The membranes were then incubated with horseradish peroxidase-conjugated secondary antibody and analyzed using ECL detection kit (GE Healthcare) and chemiluminescence imaging (LAS-1000) procedure. Immunoresponse linearity was determined by a series of sample dilutions in the range of 0.05–2.0 µg chlorophyll. Quantification of the immunoblots was done using Fujifilm LAS-1000 software.

### ProQ Diamond and Sypro staining

Proteins from thylakoids containing equal amounts of chlorophyll were separated by SDS-PAGE and stained with ProQ® Diamond phosphoprotein gel stain and SYPRO® Ruby protein gel stain according to manufacturer's instructions (Molecular Probes, Eugene, OR, USA). The gels were fixated in 50% methanol with 10% acetic acid for 30 min, the solution was changed and the fixation continued overnight, followed by washing 3×10 min in MilliQ water. Immediately prior to use the ProQ Diamond stain was equilibrated to room temperature and vigorously mixed. Gels were stained for 90 min in darkness, followed by destaining three times for 30 min with 4% acetonitrile in 50 mM sodium acetate pH 4.0, and finally washed two times in MilliQ water prior to scanning on a FLA-5100 imaging system at 532 nm. The gels were then immediately put in Sypro Ruby total protein stain and incubated over night followed by two washes in 10% methanol, 7% acetic acid and finally rinsed twice with MilliQ water. Total protein was detected on FLA-5100 imaging system at 473 nm.

### Characterization of protein phosphorylation by mass spectrometry

The thylakoids isolated from the wild type or *stn8*, *stn7*, *stn7stn8* mutant plants were resuspended in 25 mM NH_4_HCO_3_, 10 mM NaF to a final concentration of 2.5 mg of chlorophyll/ml and incubated for 3 h at 22°C with a sequencing grade-modified trypsin from Promega (Madison, WI, USA) at 5 µg of enzyme/mg of chlorophyll [22]. The peptides cleaved by trypsin were separated from the thylakoid membranes by centrifugation at 100 000 g for 30 min. The peptides were chromatographically separated using Agilent 1100 HPLC system with the flow splitter and analyzed by electrospray ionization MS in positive ionization mode using the ion trap “HCTultra PTM Discovery System” (Bruker Daltonics, Bremen, Germany). A C18 reverse phase column (5 µm; 0.3×150 mm) and a flow rate of 7 µl/min were used. A gradient of 0.1% formic acid in water (A) and 0.1% formic acid in acetonitrile (B) was distributed as follow: 0–5% B in first 30 min; 5%–15% B in 30–70 min; 15%–30% B in 70–100 min; 30–40% B in 100–120 min; 40–70% B in 120–150min and 100% B in 150–170min. The automated online tandem MS analyses were performed using collision induced dissociation of peptide ions.

### Controlled protein dephosphorylation

Controlled dephosphorylation of the peptides derived from thylakoids by trypsin treatment, as described above, was performed using alkaline phosphatase at a concentration of 100 milliunits/µl (Phosphatase alkaline, Sigma) in a buffer containing 25 mM NH_4_HCO_3_ (pH 8.0). The aliquots were taken at 0, 10, 20 and 40 min time points and dephosphorylation reactions were stopped by 1% formic acid (pH 4.0).

### Spectroscopic measurements

The spectroscopic measurements were performed using a PerkinElmer Lambda 25 spectrophotometer. The data were sampled at 2-nm intervals in the 400–750 nm range. Thylakoid membranes were resuspended at 0.0125µg chlorophyll/µl in a buffer containing 100 mM sorbitol, 25 mM Tricine (pH 7.8), 5 mM MgCl_2_, 10 mM KCl, and 10 mM NaF, which was used to preserve the native conformation of the membranes. For titration studies with different concentrations of MgCl_2_ the membranes were resuspended in the same buffer without MgCl_2_ or with MgCl_2_ at the corresponding concentration and incubated on ice for 5 min with occasional careful mixing of the solution. For pigment analysis the same membranes were dissolved in 80% acetone at 0.0125µg chlorophyll/µl.

## Supporting Information

Figure S1 Thylakoid protein phosphorylation during the photoperiod. Immunoblot of SDS-PAGE separated thylakoid proteins with anti-phosphothreonine antibody from Zymed Laboratories. Positions of the phosphorylated thylakoid proteins and of the molecular mass markers are indicated. Thylakoids were prepared from leaves of the wild type plants harvested at the indicated time points during night or day (4, 7, 19 are the hours of the dark phase and 10, 13, 16 are the hours of the light phase of the photoperiod).
(0.88 MB EPS)

Figure S2 Phosphorylation of thylakoid proteins during prolonged darkness or during dark/light transitions not related to photoperiod. Thylakoid membrane proteins from wild type plants were separated on SDS-PAGE and immunoblotted with anti-phosphothreonine antibody. A and B, thylakoids were isolated from leaves exposed for 4 hours to normal light of 120 µmol photons m-2 s-1 (Light) or for 4, 16, 20 and 24 hours of darkness, as indicated. A, immunoblot with anti-phosphothreonine antibody from Zymed Laboratories. B, immunoblot with anti-phosphothreonine antibody from Cell Signaling. Lower panels in A and B demonstrate immunoblots of the same membranes with antibodies against the CP43 and D1 proteins, correspondingly. C, thylakoids were isolated from the wild type plants exposed to normal light for 3 hours (Light), then incubated for 4 hours in darkness and re-exposed to normal light for 1 or 2 hours, as indicated. Thylakoid membrane proteins were separated on SDS-PAGE and immunoblotted with anti-phosphothreonine antibody from Zymed Laboratories.
(1.18 MB EPS)
